# Model selection in preclinical nucleic acid therapeutics research

**DOI:** 10.1038/s42003-026-09650-7

**Published:** 2026-02-09

**Authors:** Peter L. Oliver, Alyssa C. Hill

**Affiliations:** 1 MRC Nucleic Acid Therapy Accelerator (NATA), Research Complex at Harwell, Harwell Science and Innovation Campus, Oxford, UK; 2 Department of Paediatrics, Institute of Developmental and Regenerative Medicine (IDRM), University of Oxford, Oxford, UK

**Keywords:** Genetics, Molecular biology, Gene regulation

## Abstract

Nucleic acid therapeutics (NATs) are a maturing drug class with many active clinical trials and a growing number of approvals. For NATs such as antisense oligonucleotides (ASOs) and small interfering RNAs (siRNAs), a major hurdle during the research and development phase lies in selecting preclinical model systems with meaningful readouts on molecular and phenotypic efficacy. Key questions include: Which in vitro models are best positioned to quantify NAT activity and identify hits? In advancing a NAT from in vitro to in vivo studies, when is it appropriate to employ a surrogate or humanize a target locus; conversely, when is it appropriate to rely solely on human-derived cells? In this review, we will introduce and critique current approaches to ASO and siRNA preclinical efficacy studies and consider future advances in this fast-moving therapeutic area.

## Abstract (web summary)

This Review examines experimental models for evaluating nucleic acid therapeutics, emphasizing the need for effective preclinical models to assess molecular and phenotypic efficacy at each stage of development.

## Introduction

Genetic diseases are caused by changes at the DNA level. Over 6600 single gene disorders—together affecting over 4600 genes—have been identified to date^1^. Nucleic acid therapeutics (NATs), including antisense oligonucleotides (ASOs) and small interfering RNAs (siRNAs), are drugs capable of addressing genetic defects by modulating gene expression, usually at the RNA level^2,3^. Multiple ASOs and siRNAs are now approved for both rare (e.g., spinal muscular atrophy) and more common indications (e.g., cardiovascular disease)^4,5^. Indeed, decades after their discovery, and fueled by significant advances in oligonucleotide chemistry and delivery, antisense- and RNA interference (RNAi)-based technologies are now in a phase of clinical productivity^5,6^.

By contrast to “traditional” (e.g., small molecule) drugs, NATs bind and regulate their targets according to the rules of Watson-Crick hybridization; in other words, they are programmable^2,3^. To be used as drugs, NATs must be chemically modified, and certain chemical classes of NATs are now well established in the field^7,8^. This means academic and industry teams alike can design, source, test, and iterate on a library of NATs in a relatively short timeframe. Unlike for small molecules, years of extensive medicinal chemistry optimization are not necessarily required to obtain active compounds, which effectively turns traditional drug development on its head^9,10^. Therefore, for this class of drugs, the salient question is usually not: Can an active compound be generated against a given target? But rather: Which metrics of activity are meaningful? In other words, where is it best to invest time and resources in NAT preclinical development?

An essential step on the path of any drug from the bench to the clinic is an evaluation of its safety and efficacy in model systems, including cell and animal models^11^. Owing to the mechanisms of action of NATs, an accurate measure of efficacy demands alignment between the sequence of a NAT and its target: Even a single nucleotide mismatch is sufficient to alter or abolish activity^2^. This ability to discriminate target sequences is fundamental to the ASO and siRNA modalities. However, it also necessitates special care in preclinical model selection. For example, an animal model should share the target sequence with humans; if it does not, then one must either change the drug or change the model. When should one employ a surrogate NAT? When should one make a new, humanized animal model? Alternatively, when should one utilize in vitro models to the exclusion of in vivo models?

In this review, we outline ASO and siRNA preclinical assessment strategies and discuss their practicalities and limitations (summarized in Box 1). Because efficacy testing approaches vary along the preclinical path, our review is structured to reflect a typical NAT development pipeline. We begin by introducing the various in vitro screening methods at a molecular level, followed by disease-relevant phenotypic or pathway modulation in more complex cell models, and finally examine the options for in vivo modelling in a range of genetically modified (GM) systems. Looking ahead, we also consider future opportunities and challenges for these therapeutics. Although they constitute key aspects of NAT preclinical development, an in-depth look at NAT chemistry, delivery, and toxicity is beyond the scope of this review; instead, we direct the reader to recent comprehensive summaries^5,12–17^.

Box 1 ▓ **Model considerations**

**In vitro systems:**

- Genotype and expression of the target transcript and isoforms
- Translational relevance of cellular phenotypes and screening assays

**In vivo systems:**

- Conservation of the target sequence, transcript(s), and isoform(s) across species
- Availability of a well-characterized model for testing a surrogate NAT
- Resources and time to generate a humanized model
- Deciding whether and how much to humanize
**Technical challenges:**
- Delivery of ASOs and siRNAs to specific cell types and tissues
- Differences in transcriptional regulation among model systems
**Future directions:**
- Expanding the chemistry and conjugate ‘toolkit’ to improve safety and efficacy
- Developing more sophisticated in vitro models to replace animals
- Training AI models on large, homogenous datasets to facilitate drug development

## In vitro assays—molecular efficacy

### Transcript knockdown

**Fig. 1:**
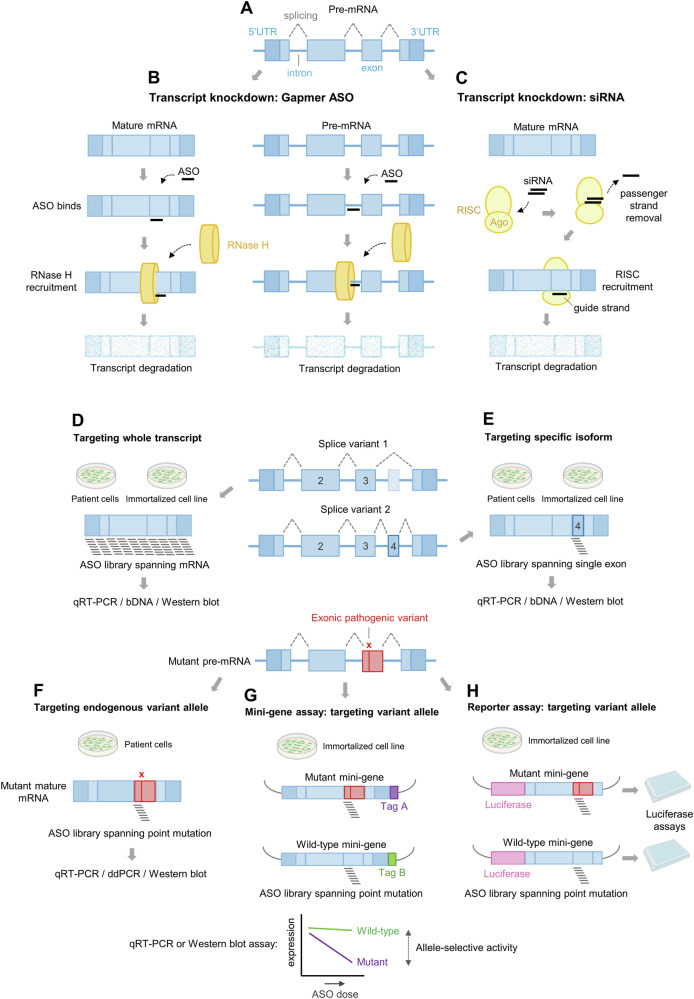
Cellular efficacy assays of target transcript knockdown using ASOs or siRNAs. **A** Structure of a typical human protein coding gene with 5’ and 3’ untranslated regions (UTRs), introns and exons. Splicing of the pre-mRNA (dashed lines) generates a mature mRNA. **B** Transcript knockdown by a gapmer ASO. The recognition of a target mature spliced mRNA (left) or pre-mRNA (right) occurs according to the rules of Watson-Crick hybridization. RNase H recognizes the DNA:RNA heteroduplex and cleaves the target RNA. In turn, the level of any encoded protein is reduced. **C** Transcript knockdown by an siRNA. A double-stranded siRNA is processed by the RNA-induced silencing complex (RISC), and the passenger (or sense) strand is released. The guide (antisense) strand guides RISC to the target RNA, which is cleaved by an Argonaute (Ago) protein. In turn, the levels of any encoded protein are reduced. **D**, **E** Knockdown of an endogenous transcript in patient cells or an immortalized cell line. **D** An ASO library is designed to span every nucleotide of splice variant 1 mature mRNA, although each ASO will also target the shared exons in splice variant 2. **E** To target splice variant 2 selectively, ASOs are designed against a single, unique exon. **F–H** Selective knockdown of a pathogenic variant allele. **F** ASOs spanning the mutated nucleotide position are screened in patient cells carrying the variant. **G** Mini-gene plasmids are introduced into immortalized cells; the co-expression of mutant and wild-type cDNAs that are differentially tagged allows for the relative quantification and thus determination of ASO allele selectivity. **H** In a reporter assay, the mutated target and a reporter (e.g., luciferase) gene are expressed in tandem. ASO activity on the target is quantified by monitoring the reporter’s activity (e.g., luciferase activity). A wild-type reporter construct can be assayed independently to generate comparative data on allele-selectivity. Some images created in BioRender; https://BioRender.com/lcouff4.

NATs can be used to knock down a target transcript by recruiting RNase H to cleave pre-messenger RNA (pre-mRNA) or mature mRNA in the nucleus or cytoplasm (‘gapmer’ ASOs, Fig. 1B), or by associating with the RNA-induced silencing complex (RISC) to cleave mature mRNA in the cytoplasm (siRNAs, Fig. 1C)^2,8,18^. This strategy is useful for, for example, reducing levels of a pathogenic, gain-of-function variant. Given the sequence-driven specificity of NATs, a library of gapmer ASOs or siRNAs can be designed to span the entire length of an RNA sequence, target a specific region in that sequence (e.g., exon or isoform), or knock down one allele with the aim of leaving the other intact (Fig. 1D, E)^19,20^. Activity can be measured on an endogenously expressed target in immortalized or patient-derived cells or, alternatively, on an exogenously supplied template. Each of these options is discussed in greater detail below.

Immortalized cell lines (e.g., HeLa, HEK293) that endogenously express the target of interest are a readily available, user-friendly option for comparing efficacy among compounds. Using this approach, large NAT libraries can be screened under lipofection, electroporation, and/or free uptake (‘gymnosis’) conditions at medium-to-high throughput^21,22^. Assisted uptake methods such as lipofection and electroporation largely control for differences in cellular uptake and intracellular trafficking among compounds, which enables head-to-head comparisons of activity independent of these factors. On the other hand, activity measured under gymnosis conditions may be more indicative of activity in vivo^21,23^. Efficacy can be measured at the RNA level using techniques such as quantitative (q)RT-PCR or branched DNA (bDNA)-based assays such as QuantiGene^24,25^ (Fig. 1D, E). Crucially, one should consider both gene *and* isoform expression: NATs directed to a part of the gene that is not included in the predominantly expressed isoform will have no or low efficacy^26^. Gene expression database searches (e.g., Genotype-Tissue Expression Portal (GTEx)^27^ or The Human Protein Atlas^28^) and RNA sequencing, including long-read analysis^29^, can help confirm the expression and isoform distribution of the target in the selected cell line. Western blotting is commonly used to assay knockdown at the protein level, although antibodies that detect specific isoforms are not always available^30^.

Patient-derived cells that endogenously express the target of interest offer an opportunity to assay NAT activity in the context of an individual’s genome, which is not possible using standard immortalized cell lines. Because early-stage NAT screening often begins with patient-derived fibroblasts or artificially immortalized patient-derived cell lines for convenience, target transcript expression should be confirmed in these cells initially^31^. Such cells are a valuable resource for studies on, for example, allele-selective NATs that are directed to a pathogenic variant or disease-associated single nucleotide polymorphism (SNP) (Fig. 1F). Genomic sequencing is also important to identify additional SNPs in the transcript that might influence NAT activity; if unexpected variants are missed, then NAT designs may be suboptimal. On the other hand, there are situations in which such variants may be exploited to achieve allele selectivity. This approach has been investigated for dominant-negative disorders such as Huntington’s Disease (HD), where SNPs or insertion-deletion (indel) polymorphisms associated with the expanded CAG trinucleotide repeat were targeted to silence the pathogenic allele specifically^32–34^. In these cases, developing an assay to distinguish knockdown on the pathogenic allele from knockdown on the non-pathogenic allele is essential. In cells that are heterozygous for a variant, quantifying the differential expression of the two alleles is possible using methods that exploit the variant itself, such as digital droplet PCR (ddPCR)^35^. Alternatively, on-target and off-target knockdown can be measured in two different cell lines, each homozygous for a variant^35^.

When a target transcript is expressed only in a specific cell type or under complex, disease-associated conditions^36^, an alternative strategy is to introduce the target into a cell line of choice as an exogenous template for screening. For example, DNA encoding the target RNA can be cloned into a vector to generate a ‘mini-gene’ plasmid that is transfected into cells for expression; NATs acting on the exogenously supplied template can be identified by qRT-PCR or Western blotting. For studies on allele-selective NATs, allele selectivity can be assayed by co-transfecting plasmids, one representing each allele and each with a different terminal tag, to enable quantification of differential expression by qRT-PCR or Western blotting (Fig. 1G). However, the secondary structure of the RNA and the level of expression of the transcript from a mini-gene may vary compared to assay systems in which the target is expressed endogenously^37^. Therefore, such workflows are valuable for measuring comparative efficacies among compounds but may not be indicative of natural target engagement.

Another approach to screening on an exogenous template is a reporter assay (Fig. 1H). Recent examples of this workflow cloned DNA encoding short target RNA regions (e.g., ~30–650 bp) into a luciferase-expressing vector^34,38,39^. The plasmid and NAT are transfected into cells, and at the assay endpoint, luminescence reports on the knockdown of a fusion transcript. The use of vectors encoding two luciferases—one for targeting and one for normalization—helps to control for variations in transfection efficiency and cell viability across experiments^39,40^. One advantage of this approach is that it can facilitate medium- to high-throughput screening of multiple haplotypes^34^. Important limitations, however, include its reliance on a proxy (i.e., luciferase enzyme activity) for the target RNA level as well as the fact that the cloned target region may lack potentially important sequence context, and the fusion transcript may adopt a secondary and tertiary structure that is distinct from an endogenously expressed gene. Indeed, a recent study of over 1000 siRNAs illustrates that data from reporter assays should be corroborated with data measured on an endogenously expressed RNA^37^.

### Splice modulation

**Fig. 2:**
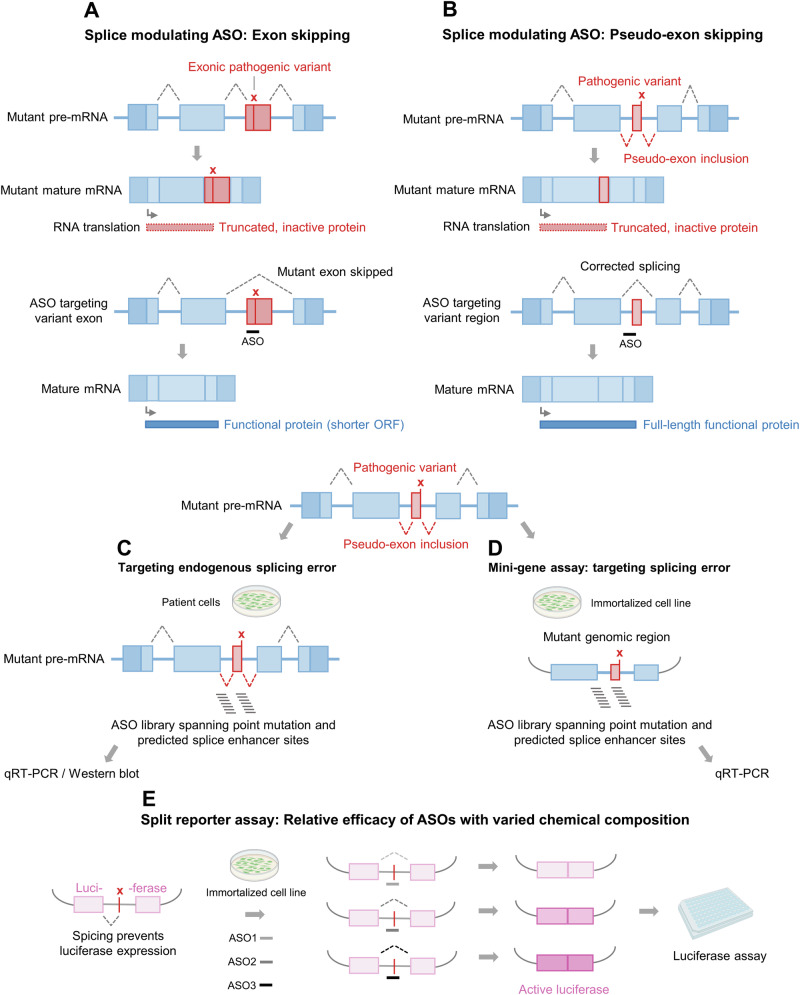
Cellular efficacy assays of splice modulation using ASOs. **A** Exon skipping via an ASO. A pathogenic loss-of-function mutation results in the introduction of a new stop codon in one exon (red), and a truncated protein is produced. Alternatively, an ASO binds to the mutant pre-mRNA, sterically blocking the normal splicing factors, and the mutant exon is skipped. The mature mRNA remains in-frame, and the open reading frame (ORF) is translated into a shortened, but functional protein. **B** Pseudo-exon skipping via an ASO. A pathogenic intronic mutation creates a new splice acceptor site that results in the inclusion of a pseudo-exon (red) into the mature mRNA. The pseudo-exon introduces a stop codon, resulting in a truncated, non-functional protein. Steric blocking of a splice enhancer region using an ASO, downstream of the mutation site, prevents pseudo-exon inclusion and rescues normal splicing and protein translation. **C**, **D** Rescue of incorrect splicing driven by a pathogenic variant; here, the mutation creates a new splice acceptor site and inclusion of a pseudo-exon (red). ASOs are typically designed over the mutation but also at proximal predicted exonic splice enhancer sites. **C** If the aberrant isoform is detectable, assays to measure splice correction can be carried out in patient cells. **D** Alternatively, assays to measure splice correction can be carried out in immortalized cells using a mini-gene plasmid that expresses the relevant intron and exons. **E** Quantifying splice modulation. Variations of chemistry on a fixed ASO sequence can be compared for the ability to repair mis-splicing of a split luciferase gene by measurement of luciferase activity. Some images created in BioRender; https://BioRender.com/lcouff4.

ASOs that do not recruit RNase H, such as those containing all ribose-modified nucleotides (e.g., 2’-*O*-methoxyethyl (2’-MOE)), can modulate the splicing of transcripts by steric hindrance of the RNA splicing machinery (‘occupancy only’ ASOs, Fig. 2A, B)^2,8^. These ASOs are useful for, for example, correcting disease-associated frameshifts that cause loss-of-function effects (Fig. 2A) or skipping pathogenic, variant-containing exons (Fig. 2B). Predictive tools can help identify the target regions for these ASOs^41^, as the regions may be distant (in sequence space) from a pathogenic variant^42^ or exon that needs to be skipped^43^.

Patient-derived cells are commonly used to evaluate splice modulatory ASO libraries, because successfully rescuing an RNA species and its encoded protein’s function may depend on context-specific pre-mRNA processing events^23,43^ (Fig. 2C). Screening is typically carried out by RT-PCR or qRT-PCR to quantify the ratio of correct to aberrant transcript^44^. Additionally, nonsense-mediated decay (NMD) inhibitors, such as cycloheximide, are useful tool compounds in situations where transcripts from cryptic splicing events are transient. These compounds can improve the sensitivity of screening assays_,_ but they also create an artificial cellular environment that might influence cell viability or cause dysregulation of gene expression that is unrelated to the activity of the ASO itself^23,45^.

A different approach is to generate a plasmid encoding a limited region of the pre-mRNA target (Fig. 2D). However, as suggested above, endogenous splicing defects can be difficult to model. The cloned region must maintain the desired splicing activity that occurs in patients, and this must occur in the cell line used to express the construct, neither of which should be taken for granted^46–48^. For example, only three of 12 predicted intronic ATP-binding cassette, sub-family A4 (*ABCA4)* pathogenic variants in the context of Stargardt Disease showed aberrant splicing in HEK293T cells expressing the cloned pre-mRNA region^49^. In one example of an ASO screen, a genomic region spanning exons 3–5 of the human ferrochelatase gene was cloned into a mammalian expression vector, including a SNP in intron 3 that promotes aberrant splicing and contributes to the disease erythropoietic protoporphyria (EPP). The resultant plasmid was used to generate two stable cell lines modeling the EPP-associated splicing defect. In turn, the cell lines were used to screen splice-switching ASOs and peptide-ASO conjugates directed to the SNP, some of which were able to increase the ratio of correct to aberrant transcript^50,51^. Of note, the size of the cloned sequence may influence NAT efficacy. In a recent study, data generated using a genomic clone flanking a single exon of the  *ABCA4* locus suggested that almost all ASOs tested were highly active. Interestingly, a longer clone spanning five exons could better delineate between NAT candidates, and these graded efficacy data were later recapitulated in patient-derived cells^52^.

Related but distinct are engineered cell lines that stably express an interrupted luciferase pre-mRNA; here, ASOs that induce splice correction generate a functional reporter protein and increase luminescence^53,54^ (Fig. 2E). Typically, the ASO sequence is optimized and fixed, but other features are varied, such as their ribose, base, or backbone chemistries or their terminal or internal conjugations^55^. The most commonly used model is HeLa pLuc/705, a cervical cancer cell line^56^. Other pLuc/705 lines have been generated from muscle, neuron, liver, and bone cell lineages, and studies in these cell lines have shown that cell type influences NAT efficacy^53^. These systems can be valuable for medium- to high-throughput comparative screening of new ASO chemistries and conjugates, but they are still affected by the technical caveats discussed above for reporter assays^37^. They are also limited by the single ASO sequence required to drive the specific splicing event that is quantified.

### Assay considerations

In many cases, designing and executing an assay to measure the in vitro efficacy of a NAT may prove straightforward. However, it is important to note that in vitro efficacy is not absolute and will depend on many factors, including cell type and doubling rate, the dose of the NAT, the duration of treatment, and the level of productive uptake and intracellular trafficking of the ASO or siRNA^21,57^. For example, a target transcript knockdown level of 80% cannot be achieved from bulk cell qRT-PCR if only 50% of the cells take up sufficient quantities of the NAT for it to be active. Conversely, when screening a NAT library, care must be taken to avoid ‘floor effects’. Here, dosing regimens are such that a considerable proportion of the NATs being screened reach the same maximal efficacy level. Such regimens, therefore, limit the opportunity to discriminateactivity for efficacy ranking.

Additionally, as the protein translated from the target transcript typically drives the disease-associated pathway, it is important to consider that knockdown observed at the RNA level may not correlate with knockdown observed at the protein level. This apparent discrepancy may be due to the complexities of post-translational modifications and turnover kinetics of the protein, whereby feedback loops driving stabilization or targeted degradation occur irrespective of transcript availability^58^. This may or may not be overcome by increasing the time between NAT dosing and measuring protein levels, so this specific temporal relationship needs to be determined empirically. Similarly, ‘successful rescue’ of a mis-splicing event using an ASO, resulting in an increased amount of the wild-type transcript (e.g. Fig. 2B), may not always proportionally rescue the target protein. Many biological events may contribute to this phenomenon, including the failure of the rescued RNA species to be suitably polyadenylated or trafficked for translation^23^. Furthermore, recent work has illustrated that the perseverance of NAT binding to the target RNA can result in amplification bias during RT-PCR assays, resulting in a false impression of splicing rescue efficacy^59^. Finally, protein quantification can be limited by issues around the specificity and sensitivity of the available antibodies for Western blotting or immunocytochemistry assays; ideally, more than one reagent or method is used to confirm NAT efficacy at the protein level^29,30^. However, as demonstrated recently by Klein et al., when well-characterized antibody reagents are in hand, the efficacy of NATs targeting multiple, complex protein isoforms can be quantified accurately^60^.

## In vitro assays—phenotypic efficacy

As outlined above, early steps in the NAT development pipeline include quantifying target RNA and protein levels to provide evidence that a NAT is engaging its target. Following on from this initial screening phase, measuring the function of the target protein and/or proteins downstream in a biological pathway can provide supporting evidence for efficacy and demonstrate that disease-relevant mechanisms are being modified as required by the therapeutic profile of the drug. This is particularly relevant for metabolic disease, as fundamental biochemical pathways are often conserved between model systems and are reproducible in vitro, such as the reinstatement of enzymatic activity^61^. For example, to test a library of ASOs designed to prevent disease-associated pseudo-exon inclusion and restore the function of the glycogen branching enzyme (*GBE1*), Thomas et al. utilized patient fibroblasts to demonstrate rescue of both the expression and activity of protein^29^. Importantly, such studies can determine not only the degree of functional protein rescue that can be obtained realistically by NATs, but also how much restored enzyme activity might be required to compensate therapeutically for disruption of the specific metabolic pathway^61^. Similarly, there may be physiological metrics of efficacy that can be quantified in patient-derived cellular systems. For example, in bronchial epithelial cells derived from cystic fibrosis patients, chloride secretion is key indicator of a therapeutically beneficial drug, and NATs that correct pathogenic cystic fibrosis transmembrane conductance regulator (*CFTR*) splicing have been identified using this assay^62^.

When the downstream pathways influenced by modulating the target are well known, an alternative approach is to engineer a cell line to report on a specific functional event. Recent examples include manipulating T-cell receptor (TCR) function using NATs. First, the lymphocyte cytosolic protein 2 (*LCP2*) gene was successfully targeted by screening an ASO library in a T-cell line^22^. Next, TCR activation was quantified in the same cell line using a fluorescent reporter knocked into to a key transcription factor (NFκB) in the known signaling pathway. Cells could be treated with a range of T-cell activators in tandem with ASO delivery, and the degree of stimulation was quantified by flow cytometry from the fluorescent reporter as a downstream output of phenotypic efficacy^22^.

Three-dimensional (3-D), patient-derived cell systems provide an option for efficacy testing when the phenotype of interest relies on cell-cell interactions or more complex physiology. These models, including multicellular organoids and assembloids, aim to recapitulate aspects of tissue interconnectivity and function and are particularly important when no suitable animal models are available^63–65^. Some early studies in the NAT field have focused on modelling the nervous system, where iPSC-derived neurons and 3-D organoids can recapitulate some disease-relevant functional phenotypes, such as seizure-like circuit activity^64^. However, these cultures are technically challenging to establish and can take many months to reach the connective maturity required to measure meaningful physiological outputs^64^. Interestingly, away from nervous system disorders, a recent study has shown how even a relatively simple organoid platform can be harnessed to screen multiple patient-specific ASOs. Means et al. focused on a small panel of robust phenotypic assays that are amenable for quantification in cardiac organoids^66^. Patient-specific ASOs, designed to prevent the individual’s aberrant dystrophin splicing, were tested in these 3-D cultures, and abnormal calcium flux and rhythmic contraction rates were restored^66^. This study illustrates that when selecting to work with complex cell models for NAT assessment, focusing on throughput and specific phenotypic readouts can be an efficient preclinical strategy.

Notably, as 3-D cellular systems become closer physiologically to tissues, they will share more of the practical challenges that influence NAT efficacy in whole organs, such as variable cell type-specific uptake and on-target activity^17^. Indeed, a molecular understanding of differential efficacy in specific cell types is still evolving^67^. For example, in a model of Timothy Syndrome using human forebrain organoids, flow cytometry was used as a general measure of fluorescently labelled ASO uptake in the model system^68^. However, phenotypic experiments were carried out with an untagged ASO, and no data regarding its spatiotemporal distribution versus on-target activity were shown. Care must be taken when interpreting such datasets, where uptake and penetration of the NAT into the 3-D structure is critical, in addition to potentially variable cell type-specific activity^69^.

## Ultra-rare and *N* = 1 disorders

Although ultra-rare and single patient (‘*N* = 1’) disorders are arguably a niche area of drug discovery, future innovation in the NAT field will continue to be shaped by ultra-rare and *N* = 1 trials. In these cases, patient-derived cells may be the only practical efficacy testing platform, and time constraints may necessitate an expedited preclinical path^70,71^. Examples to date have relied typically on NAT efficacy testing in patient fibroblasts, followed by generating evidence for functional rescue of key biochemical or cellular phenotypes^23,72^. For Milasen, the first approved *N* = 1 ASO, multiple biochemical and cellular imaging efficacy metrics were obtained from the patent’s fibroblasts to demonstrate some rescue of lysosomal storage dysfunction using the lead compound^73^. Indeed, Milasen and other NATs have gained approval with no efficacy or phenotypic modelling in any animal system, apart from limited acute toxicity studies in rodents^71,73^. This contrasts with the typical FDA drug development pipeline for a NAT, which requires animal safety testing in more than one species—including non-human primates (NHPs)—prior to investigational new drug (IND) filing and first-in-human studies^74,75^. As this important area of NAT clinical investigation expands, rapid access to stem cell-derived, physiologically relevant cell models are needed to assess efficacy and functional biology that may be cell-type specific^20^.

## In vivo efficacy

**Fig. 3:**
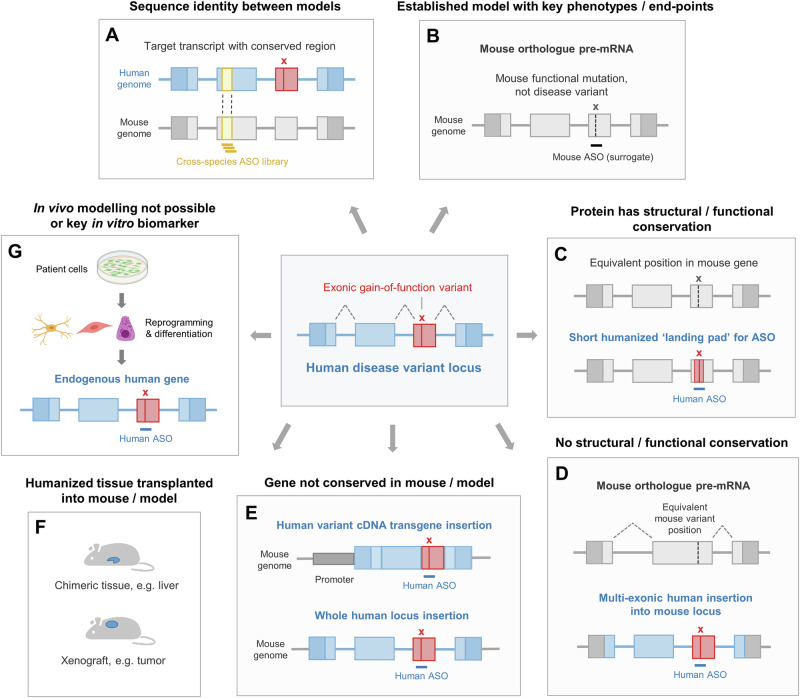
Humanization strategies for ASO efficacy testing. **A**–**E** In this example, the aim is to model a pathogenic heterozygous gain-of-function variant in the mouse and then knock down expression of the target gene. **A** Using a non-allele-selective approach, a region of the mRNA that is conserved between human and mouse can be used to design an ASO library potentially active in both species. **B** Using an allele-specific approach, proof-of-principle mechanistic hypotheses can be tested and/or well-established phenotypes can be modulated using a mouse-specific, surrogate ASO. **C** If the human and mouse proteins are similarly organized genomically and structure and function are conserved, humanizing only a minimal region may be required for testing ASO efficacy. **D** If the mouse protein is structurally or functionally diverse from the human protein, it is possible to integrate many exons of the variant human gene into the mouse genome; in this example, mouse UTRs are maintained to preserve species-specific regulation. **E** If there is no equivalent gene in the mouse genome, options include inserting a cDNA transgene or entire human gene, with or without flanking regulatory regions, for testing ASO efficacy. **F** Transplantation of human-derived tissue, such as a tumor, or chimeric tissue populated by human cells, such as the liver, can facilitate testing ASO efficacy. **G** Due to practical reasons, time constraints, or the conservation of key cellular disease-associated pathways, patient-derived in vitro models are an alternative or parallel model system. Some images created in BioRender; https://BioRender.com/lcouff4.

In vitro models provide multiple options for NAT efficacy testing, while advanced cell models—often patient-derived—are valuable for assessing engagement with more complex cellular pathways. Moving to in vivo models allows increasingly sophisticated metrics of phenotypic efficacy to be measured, in particular when key aspects of disease biology cannot be captured in vitro, such as multisystem pathologies and behavior. However, the conservation of the underlying biology between species must be considered. While some physiological features of animals, such as rodents, can provide reliable and predictive phenotypic readouts, fundamental aspects of many human diseases—including complex gene-environment interactions and ageing—are far more challenging to model^76,77^. Moreover, when the intention is to develop a human-targeting ASO or siRNA, a key decision point for both drug design and in vivo efficacy model selection is whether there is potential for alignment between the human NAT sequence and the model system’s genome. Focusing only on sequences conserved between species might expedite the design and screening process, but this may come at the expense of efficacy. Alternatively, humanizing the model in some way—such as introducing a portion of a human gene into the model’s genome—is more time-consuming, but allows the same NAT to be used in cross-species efficacy studies. Thus, there are multiple experimental options available, each providing a different balance between time, cost, and predictive, translational relevance; summary exemplar decision points for in vivo efficacy modelling are outlined below and in Fig. 3.

### NAT sequence conservation or adaptation

The sequence of an mRNA will vary considerably among species, yet given that NATs typically target only short regions of 16–30 nucleotides^8^, every human target will likely have some conserved sequences of this length. Therefore, NAT library design can be focused solely on these areas (Fig. 3A), with the assumption that the same lead molecule can be taken through all stages of preclinical development. Indeed, there are a small number of examples where a lead NAT was designed against a conserved sequence; these include siRNAs cross-reactive to Janus kinase 1 (*JAK1*) in humans and a variety of other species^78^. Here, the aim was to exploit the established models of inflammatory skin disease in rodents, pigs, and ultimately NHPs to accelerate the preclinical development path^78^. However, it is important to note that this approach may severely restrict design space and compromise NAT potency, with potentially more efficacious and safer compounds targeting human-specific sequences left untested.

A second, related strategy is to convert a human-directed compound to one targeting the equivalent region in the model. However, a recent study adapted human siRNA sequences to match the same target region in another species; in many cases, the siRNAs produced by this ‘mismatch conversion’ approach were less efficacious than the siRNAs identified via a species-focused screen^79^.

### Surrogates

As an alternative to finding evolutionary conserved target sites, library design can be focused on the model’s genome alone, generating a species-specific, or surrogate, NAT. For example, prior to the advent of NATs, many GM mouse models were established with ‘mouse limited’ modifications where only nucleotides encoding a single disease-causing amino acid were changed, but not alongside any adjacent human sequence^80^. As such, targeting these models requires a surrogate NAT (Fig. 3B). However, if an established, well-characterized disease model is available, data from surrogates allows one to take advantage of robust and reproducible efficacy metrics and biomarkers. Here, the translational or mechanistic relevance of a study in such a model should be weighed against the resources required to design and screen for a surrogate NAT. Examples include a mouse model carrying a well-studied gain-of-function *SCN2A* sodium channel variant, which demonstrated that mouse-specific ASO-mediated knockdown of both alleles could mitigate seizures and epilepsy-associated cognitive and behavioral comorbidities^81^. These proof-of-concept phenotypic data paved the way for clinical development, with recent successful outcomes from the first human-specific ASO treatment in a patient with *SCN2A*-associated developmental and epileptic encephalopathy^82^. The major disadvantage of using a surrogate NAT is that it has a different sequence to the human-directed NAT of interest, which can influence pharmacokinetic (PK) parameters and limit allosteric projections. However, exaggerated on-target toxicity effects can still be examined as part of an overall safety package^12^. Surrogates also have broader utility: A well-studied surrogate ASO or siRNA, such as one targeting Huntingtin (*Htt*), Cyclophilin B (*Ppib),* or the non-coding RNA Metastasis Associated Lung Adenocarcinoma Transcript 1 (*Malat1*)^83–85^, can be valuable for evaluating the pharmacokinetic/pharmacodynamic (PK/PD) properties of NATs with new chemistries and conjugations.

Notably, however, the human target may not have a non-human orthologue for surrogate NAT targeting, as evolutionary variations in gene size and isoform composition and the presence of multiple species-specific paralogues are common^86^. Furthermore, the biological mechanisms that underpin cellular and tissue RNA modulation, including those that influence NAT activity, are often divergent between species; these include regulatory networks driven by transcription factors^87,88^. In summary, species-specific, surrogate NATs are valuable for proof-of-concept studies in wild-type or GM systems, but it should not be assumed that their functionality will be reflective of the human-targeting counterparts.

### Humanization

A significant proportion of in vivo NAT efficacy modelling is carried out in mice, a mammalian system with a well-established toolkit for genetic manipulation^89^. Undoubtedly, the engineering of small (e.g., single nucleotide) and large (gene replacement) genomic alterations in the mouse has been revolutionized by CRISPR/Cas9 methods (reviewed in ref. ^89^), yet diligence around model design and characterization is essential. Humanized models require a considerable investment of time and resources. Therefore, the choice to humanize will be informed by the confidence in the lead compounds identified during in vitro screening and the power of the mouse to demonstrate quantifiable, disease-relevant phenotypes or pathologies that test NAT activity against the specific human target sequence. Furthermore, before embarking on a new in vivo experiment, it is important to refer to databases that catalogue GM resources to ensure that a suitable model is not already available^90^.

Once a decision to humanize has been made, a key follow-on question is how much to humanize? As discussed above, NATs have the ability to discriminate between target sequences that differ by a single nucleotide. Humanization of a target region may require multiple sequence adaptations to create a ‘humanized landing pad’ for efficacy testing^39^ (Fig. 3C). However, even such minor sequence changes may alter the secondary structure of the model’s RNA. This in turn, may affect the target’s accessibility to the NAT, although methods to reliably predict this phenomenon are not yet established^44,91,92^.

The recent transition from exome to whole genome sequencing in clinical genetics has enabled the identification of a large number of novel, disease-associated intronic variants predicted to influence gene function^93^. In such cases, humanization requires a larger genomic region to be replaced or engineered to generate a model system for NAT testing (Fig. 3D). Recapitulating the functional effects of such variants in the mouse is not always straightforward, however. To model Leber congenital amaurosis (LCA), a splice donor site mutation in *CEP290* was engineered into mice using a 6.4 kb human region, but this unexpectedly resulted in very low expression of the key disease-associated isoform, precluding effective NAT screening^94^. This study demonstrates the subtleties of species-specific recognition of splicing, which needs to be considered and investigated empirically.

To circumvent species divergence of transcript organization and even protein structure, a third option is to replace an entire mouse gene with a human copy or add one or more additional human genes into the mouse genome for NAT targeting (Fig. 3D). For example, to target the human Ataxin 3 gene (*ATXN3*) as a model for spinocerebellar ataxia type 3 (SCA3), two independent mouse transgenic lines were tested: one spanning the entire human *ATXN3* locus and the other an *ATXN3* cDNA transgene^95^. Interestingly, ASOs active against the locus-derived mRNA were inactive against the *ATXN3* cDNA transcript; this study exemplifies how differences in RNA processing dynamics and isoform expression influence NAT efficacy^95^. For whole gene humanization studies, the genomic context of the insertion is critical. The choice is whether to maintain the endogenous mouse regulatory regions, such as the UTRs, or to humanize a larger genomic region that includes the equivalent human flanking sequence (Fig. 3E)^96^. Importantly, UTRs and upstream DNA likely contain multiple sites for transcriptional modulatory factors that can be highly species-specific and cell-type defined; therefore, thoroughly testing the spatiotemporal expression of the humanized target locus at the RNA and protein level is essential^96^. In summary, genetic humanization for NAT efficacy studies requires careful consideration of the experimental hypotheses combined with diligent characterization of the resulting model at a molecular level.

### Tissue humanization

Humanization also includes the addition or replacement of human cells or tissue in an animal host, thus generating a human cell-specific environment for efficacy testing (Fig. 3F)^97^. One particularly challenging area for NAT research and development is oncology, as tumors are notoriously heterogeneous, and inter- as well as intratumor heterogeneity can impact on NAT efficacy in vivo^98^. Moreover, the use of humanized orthotopic xenograft approaches raises questions around the context of human tissue in a rodent host. For example, Ma et al. were able to test human-specific isoform-switching ASOs that were active in hepatocellular carcinoma (HCC) tumors transplanted into mouse^99^. However, there are drawbacks of mixed-species xenograft models that will influence NAT efficacy and tolerability. The requirement to utilize immunocompromised hosts will dampen toxicity-relevant immune responses, and the interaction between the human tumor microenvironment and the murine immune system will potentially influence NAT uptake and tumor penetration^100^. Therefore, testing a surrogate ASO library in a mouse model may be a valuable alternative to better model the physiology of endogenously induced tumor growth^99^. As introduced above, if no suitable animal model is available, or time constraints preclude development, patient-derived systems may be the only practical solution (Fig. 3G).

### Large animal models

Interestingly, large animals are being utilized more commonly for genetic disease modelling. Their application aims to address the physiological limitations of rodents to model human pathogenic processes, such as their small organ size and limited lifespan^101^. Indeed, the suitability of specific anatomical features often justifies the significant time and financial investment required to establish a new GM large animal model. Examples of non-human NAT surrogate testing include a humanized mini-pig engineered to carry a recurrent Duchenne muscular dystrophy (DMD)-associated exon 52 deletion^102^. These DMD pigs manifest early onset skeletal muscle degeneration, with pathological features that closely resemble the human disorder for future evaluation of in vivo exon skipping and muscle functional rescue^102^.

Retinopathies are attractive for NATs, given the opportunity for direct intravitreal (IVT) delivery to the eye. For CLN3 Batten disease, retinal degeneration is an early clinical symptom, yet mice recapitulating the most common pathogenic deletion display only limited retinal dysfunction with normal electroretinogram (ERG) outputs^103^. As the pig eye is much closer in size to the human eye, with more representative retinal cell-type composition than in the mouse, a mini-pig *CLN3* deletion mutant was generated. Crucially, this model displayed progressive loss of vision, retinal pigment epithelium dysfunction, and photoreceptor cell degeneration to better model the disease. Subsequent studies with ASOs designed to target both the pig and human *CLN3* locus demonstrated long-term rescue of ERG defects after a single IVT dose, facilitating future clinical development^103^.

With more efficient genetic manipulation methods becoming readily established outside of rodents, it will be interesting to see whether the NAT field invests more in these larger preclinical models. Considering the time and resource commitment required to generate and maintain experimental cohorts, sample sizes may necessarily be smaller than  in studies using rodents. Yet the potential to model more faithfully specific human pathogenic mechanisms, without the use of NAT surrogates in some cases, may encourage uptake^78,104,105^. Furthermore, disease compromised large animal models may provide more predictive metrics of NAT efficacy, PK, and toxicity than non-GM species^106^. Relatedly, studies are just beginning to quantify the altered PK properties of NATs in patients with organ impairment, such as renal failure^107^. The ability to model these outcomes may become more important in the future as the use of NATs in the clinic becomes more widespread.

## Future perspectives

Based on current trajectory, the future is bright for NATs. Many late-stage clinical trials are ongoing, and further approvals are imminent. Each success reinforces acceptance by patients, caregivers, and the medical profession, while simultaneously establishing the necessary development, regulatory, and ethical frameworks. Additionally, scientific innovation is beginning to meet core challenges for the field, such as delivery to extrahepatic cell types^10,11^. As *N*-acetylgalactosamine (GalNAc) revolutionized delivery to hepatocytes in the liver^7^, novel conjugate groups that can deliver NATs to other tissues are on the horizon. For example, evidence is mounting that ligands of the transferrin receptor (TfR) achieve delivery to muscle; notably, antibody-based delivery systems may require a rodent surrogate antibody to model in the mouse^108^. Meanwhile, similar TfR approaches are gaining traction in the central nervous system (CNS)^109^, and NATs of new architectures that access the CNS are advancing into the clinic^110^. Furthermore, although certain chemical classes of NATs have taken hold in the field—and benefit from significant legacy pharmacology and safety datasets^111^—novel chemistries are progressing through clinical trials^112^. When the next step-change in NAT chemistry and/or delivery is discovered, the field must be agile in developing updated preclinical safety and efficacy pipelines to expedite the clinical path.

The future of the NAT field will likely be shaped by large-scale, high-throughput methods to optimize design and potency. Artificial intelligence and machine learning (AI/ML) approaches for predicting NATs with optimal efficacy are emerging and will undoubtedly become more accepted for building predictive, optimized NAT sequence and chemistry screens^113^. This includes the retrospective interrogation of efficacy datasets that are ideally well-controlled and standardized for optimal training performance^113–115^. For example, to predict target transcript knockdown efficacy, a recent study trained a model on over 188,000 data points from gapmer ASO screens published in the patent literature^116^. Combining ASO sequence design, chemical modification pattern, cell delivery method, and dose information, the tool aims to significantly reduce the number of oligonucleotides that need to be screened to identify potent compounds^116^.

Considering the future of preclinical modelling, a desire to reduce experimental animal use, combined with recent directives from governmental funding bodies^117,118^, will in part drive the further development of more sophisticated patient-derived organoids and microphysiological systems. These models will likely accelerate NAT preclinical development pipelines, and current advances include the automation of 3-D culturing methods to enhance throughput and reproducibility^119^. Furthermore, when combined with in silico methods for modelling NATs, there is considerable potential to build improved, predictive in vitro preclinical platforms for safety and efficacy^115^. The utility and broader uptake of these technologies, however, will hinge on thorough benchmarking and an acceptance that complex in vitro systems may not always be complete replacements for animal models but rather may offer additional, complementary evidence for modulating a specific cellular phenotype^120,121^.

Here, we have reviewed and critiqued the models used in preclinical NAT development, and the key points are summarized in Table 1. We emphasize that model selection will be specific to the nature and phase of the project. It is also important to appreciate the advantages and limitations of the various systems available, as well as diligently apply experimental controls (Table 1). As introduced above, a new NAT project can yield promising preclinical outcomes in a relatively short period of time; yet careful consideration of the potential translational path is warranted. For example, pathophysiological mechanisms related to the target should be well established, and timely, targeted clinical intervention using NATs should be possible. In the early NAT screening phase, there are often tradeoffs between throughput and translational validity, but reagents and assays may be available to quantify efficacy with true mechanistic or preclinical value. This includes looking ahead to clinical biomarkers, where there may or may not be relevant proxies in model systems. Moreover, when a ‘gold-standard’ rodent disease model is available, demonstrating proof-of-concept using a surrogate NAT might be more efficient than generating a new humanized system with potentially unpredictable cross-species genetic and genomic interactions that hamper applicability^81^.

**Table 1:** Summary of NAT efficacy models and key controls that need to be considered during NAT efficacy screening

	Advantages/Disadvantages	Key controls
**Immortalized cell lines**	+ Accessibility + Ease of use + Endogenous target/native context factors + Suitable for medium- to high-throughput screening by transfection, gymnosis or electroporation - Target may not be (sufficiently) expressed - Translational relevance is not guaranteed	• Confirmation of target expression (e.g., GTEx searches, short/long-read RNA-seq)^29^ • Negative control NAT(s) (e.g., non-targeting and mismatch controls)^21,123^ • Positive control NAT(s) (e.g., to confirm cellular uptake and viability)^21^
**Mini-gene assays**	+ Accessibility + Ease of use + Suitable for medium- to high-throughput screening in immortalized cell lines via transfection - Exogenous target/no native context factors	• Confirmation that the (mis)splicing event of interest is recapitulated^52^ • Negative control NATs (e.g., non-targeting and mismatch controls)^21,123^ • Positive control NAT(s) (e.g., to confirm cellular uptake and viability)^21^
**Reporter assays**	+ Accessibility + Ease of use + Suitable for medium- to high-throughput screening in immortalized cell lines via transfection or gymnosis in stable cell lines - Exogenous target/no native context factors - Relies on a proxy (i.e., luciferase enzyme activity) for RNA target levels	• Negative control NATs (e.g., non-targeting and mismatch controls)^21,123^ • Positive control NAT(s) (e.g., to confirm cellular uptake and viability)^21^ • Measurements on a second luciferase to account for variation in transfection efficiency and cell viability^39^
**Patient-derived cells**	+ Endogenous target/patient-specific native context factors + Possible to reprogram to specific cell types - Not always accessible for practical and/or ethical reasons - Less user-friendly than immortal cell lines	• Immunocytochemistry/labelled NATs to quantify uptake and distribution^69^ • Control cell lines (e.g., parental/carrier or isogenic)^124^ • Cells from multiple patients carrying the same variant (e.g., confirm on-target/functional effects)^66^ • Multiple iPSC lines/differentiations from the same donor (e.g., to demonstrate reproducibility)^124^
**3-D cell models**	+ Better able to model complex cellular and multicellular processes than 2-D systems + Possible to study hard-to-access cell types/systems + Helps meet the demand to replace animals - Time and cost to generate complex models (e.g., assembloids/multi-tissue systems) - Suitable for low- to medium-throughput screening	• Immunocytochemistry/labelled NATs to quantify uptake and distribution^69^ • Internal control cell-type markers for culture reproducibility and comparative composition^121^ • Multiple iPSC lines/differentiations from the same donor (e.g., to demonstrate reproducibility)^124^
**Rodent models**	+ Whole mammalian system + Possible to interrogate biodistribution and toxicology + Availability of established and well-characterized disease models + Surrogate NATs in wild-type rodents enable PK/PD studies/NAT screening - Human gene/locus is not always conserved - Time and cost to generate humanized models - Physiology of rodents may not recapitulate disease/no model may be available - Class-specific toxicology profiles may differ to human - Ethical concerns	• Human wild-type knock-in transgenic line as a comparator for human variant knock-in transgenic line^125^ • Confirmation of RNA and protein expression (if applicable) and functionality of exogenous humanized (trans)gene^96^ • Quantification of NAT biodistribution versus efficacy (e.g., PET/radiolabelling, fluorescent-based imaging, mass spectrometry, immunohistochemistry, molecular methods)^126^ • Diligent experimental design and reporting^127^
**Large animal models**	+ Whole mammalian system/native context factors + Possible to interrogate biodistribution and toxicology + Anatomy and physiology can have more relevance to humans’ than rodents - Cost to generate and maintain models - Ethical concerns	• Human wild-type knock-in transgenic line as a comparator for human variant knock-in transgenic line^125^ • Confirmation of RNA and protein expression (if applicable) and functionality of exogenous humanized (trans)gene^101^

For the ASO/siRNA field to continue progressing at its current pace, advances in NAT chemistry, efficacy, delivery, and safety will be required, but also a willingness to share standardized model systems as well as preclinical data, both positive and negative^122^. Given the striking variability across the published literature, emerging recommendations around efficacy screening, alongside accurate reporting and validation of the model systems used, are vital for reproducibility in the field. This, in turn, will encourage community-wide consensus around best practice and help secure the future translational potential of safe, efficacious, and accessible NATs.

### Reporting summary

Further information on research design is available in the Nature Portfolio Reporting Summary linked to this article.

## Supplementary information

Reporting summary
